# Overexpression of MTFR1 promotes cancer progression and drug-resistance on cisplatin and is related to the immune microenvironment in lung adenocarcinoma

**DOI:** 10.18632/aging.205338

**Published:** 2024-01-02

**Authors:** Qian-Yun Li, Qiang Guo, Wei-Min Luo, Xiang-Yu Luo, Yan-Mei Ji, Li-Qiang Xu, Jia-Long Guo, Rong-Shu Shi, Feng Li, Cheng-Yi Lin, Jun Zhang, Di Ke

**Affiliations:** 1 Department of Radiology, Affiliated Hospital of Zunyi Medical University, Zunyi, Guizhou, China; 2 Department of Cardiothoracic Surgery, Taihe Hospital, Hubei University of Medicine, Shiyan, China; 3 Department of Critical Care Medicine, Taihe Hospital, Hubei University of Medicine, Shiyan, China

**Keywords:** lung adenocarcinoma, prognosis, MTFR1, drug resistance, immune infiltration

## Abstract

Objective: The roles of MTFR1 in the drug resistance of lung adenocarcinoma (LAC) to cisplatin remain unexplored. In this study, the expression, clinical values and mechanisms of MTFR1 were explored, and the relationship between MTFR1 expression and immune microenvironment was investigated in LAC using bioinformatics analysis, cell experiments, and meta-analysis.

Methods: MTFR1 expression and clinical values, and the relationship between MTFR1 expression and immunity were explored, through bioinformatics analysis. The effects of MTFR1 on the growth, migration and cisplatin sensitivity of LAC cells were identified using cell counting kit-8, wound healing and Transwell experiments. Additionally, the mechanisms of drug resistance of LAC cells involving MTFR1 were investigated using western blotting.

Results: MTFR1 was elevated in LAC tissues. MTFR1 overexpression was associated with sex, age, primary therapy outcome, smoking, T stage, unfavourable prognosis and diagnostic value and considered an independent risk factor for an unfavourable prognosis in patients with LAC. MTFR1 co-expressed genes involved in the cell cycle, oocyte meiosis, DNA replication and others. Moreover, interfering with MTFR1 expression inhibited the proliferation, migration and invasion of A549 and A549/DDP cells and promoted cell sensitivity to cisplatin, which was related to the inhibition of p-AKT, p-P38 and p-ERK protein expression. MTFR1 overexpression was associated with stromal, immune and estimate scores along with natural killer cells, pDC, iDC and others in LAC.

Conclusions: MTFR1 overexpression was related to the unfavourable prognosis, diagnostic value and immunity in LAC. MTFR1 also participated in cell growth and migration and promoted the drug resistance of LAC cells to cisplatin via the p-AKT and p-ERK/P38 signalling pathways.

## INTRODUCTION

Lung adenocarcinoma (LAC) had a high morbidity and mortality rate [1, 2]. The prognosis of patients with early LAC could be improved by surgery and neoadjuvant chemotherapy. However, despite complete tumour resection, primary and secondary drug resistance often leaded to poor patient prognosis [2, 3]. Novel targeted therapies had enhanced the prognosis of patients with LAC. However, the overall prognosis remains unsatisfactory [4–6]. Therefore, it is of great clinical and scientific significance to elucidate the unfavourable prognostic biomarkers involved in the progression of LAC.

Mitochondrial fission regulator (MTFR) 1 was a member of the MTFR family. Mitochondrial abnormality has been related to the progression of cancer and other diseases [7–10]. Zeng et al. reported that Faciogenital Dysplasia 1 (FGD1) was upregulated in hepatocellular carcinoma (HCC), and increased FGD1 expression correlated with the progression and unfavourable prognosis of patients with HCC. Moreover, knocking out FGD1 in cancer cells significantly inhibited the malignant behaviour of HCC, causing mitochondrial dysfunction and highlighting its oncogenic properties [10]. MTFR1 was a mitochondrial regulator that played a role in regulating mitochondrial fission and cell progression [11–16]. Wang et al. reported that miR-324-5p reduced mitochondrial fission, myocardial cell apoptosis and myocardial infarction by inhibiting MTFR1 [11]. Similarly, Huang et al. found that myocardial ischemia-reperfusion injury increased the expression of the long-chain non-coding RNA (lncRNA) GPR19. The inhibition of lncRNA GPR19 expression in C57BL/6 mice improved the cardiac function and reduced the apoptosis and myocardial fibrosis scar formation. Additionally, lncRNA GPR19 reduced oxidative stress and apoptosis in neonatal rat ventricular cardiomyocytes induced by oxygen-glucose deprivation/recovery by miR-324-5p/MTFR1 [14]. Wang et al. observed that MTFR1 could be used to classify tumours based on the extracapsular spread status. In cancer patients with negative lymph nodes, the overall survival (OS) of patients with extracapsular spread-positive tumours was significantly worse [15]. Consistently, Li et al. reported that MTFR1 was overexpressed in LAC and associated with unfavourable prognosis of patients with cancer. MTFR1 could enhance the proliferation, invasion, migration and glycolysis of LAC cells [16]. However, the roles and pathways of MTFR1 in the resistance of LAC cells to cisplatin remain unreported. Therefore, this study investigates the roles and mechanisms of MTFR1 in LAC progression using comprehensive analysis, and basic research to evaluate whether MTFR1 has the potential as a therapeutic marker for patients with LAC.

## RESULTS

### MTFR1 expression was increased in LAC tissues

**Figure 1:**
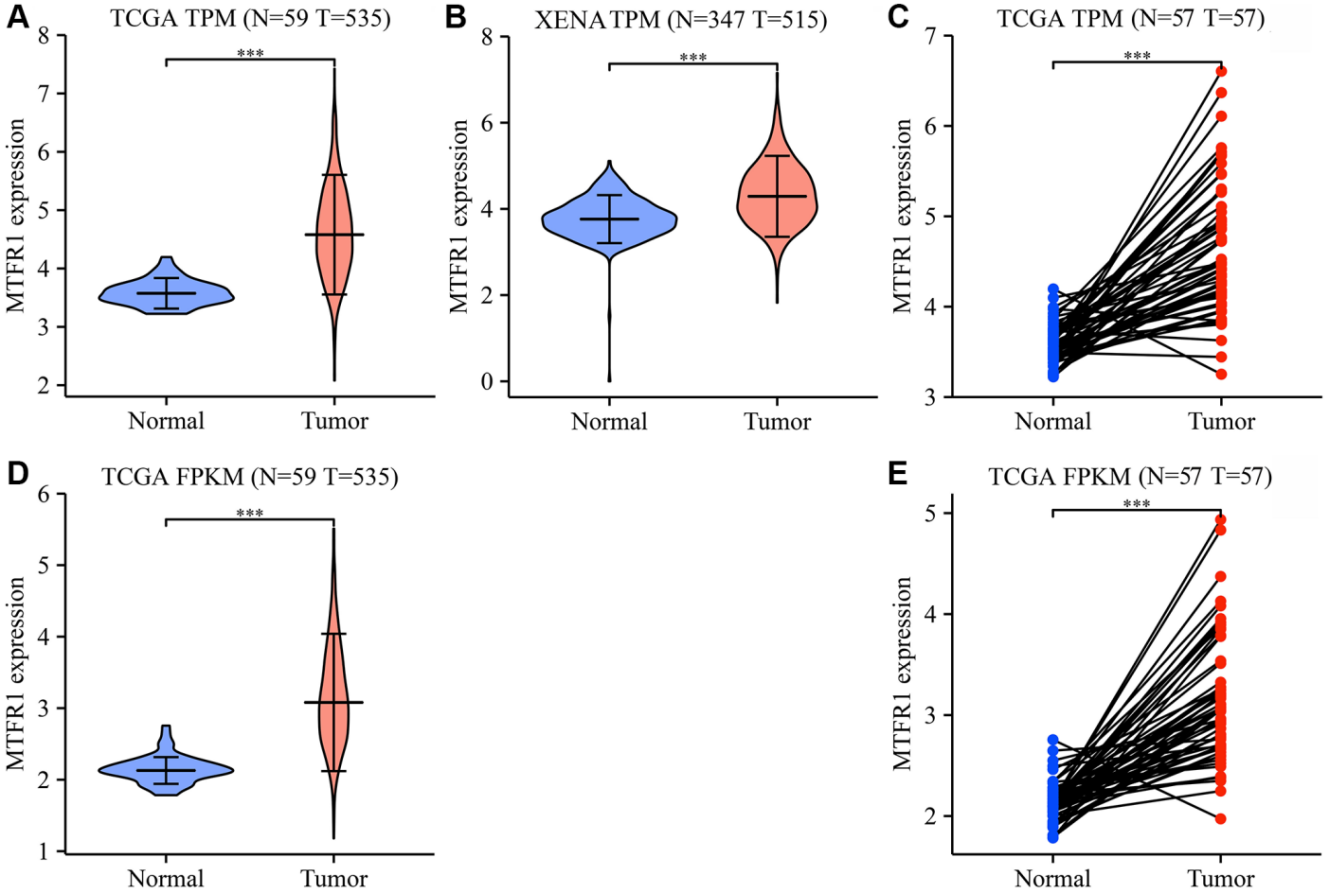
**MTFR1 expression levels in LAC tissues from the TCGA and XENA databases.** (**A**–**C**) The data of TPM type; (**D**, **E**) The data of FPKM type. Abbreviations: LAC: lung adenocarcinoma; TCGA: The Cancer Genome Atlas; TPM: transcripts per million; FPKM: Fragments Per Kilobase Million; ^***^P < 0.001.

The Cancer Genome Atlas (TCGA) and XENA database analysis revealed that MTFR1 expression was increased in LAC tissues (Figure 1 and Supplementary Figure 1). In detail, the levels of MTFR1 increased significantly in unpaired and 57 paired LAC patents from TCGA and XENA transcripts per million (TPM) type data (Figure 1A–1C), and in unpaired and 57 paired LAC patents from TCGA fragments Per Kilobase Million (FPKM) type data (Figure 1D, 1E). Additionally, MTFR1 expression levels were also significantly increased in LAC tissues based on the meta-analysis of the TCGA and Gene Expression Omnibus (GEO) databases (Supplementary Figure 1).

### MTFR1 overexpression correlated with diagnosis, sex, age, therapy outcome, smoking and T stage in patients with LAC

**Figure 2:**
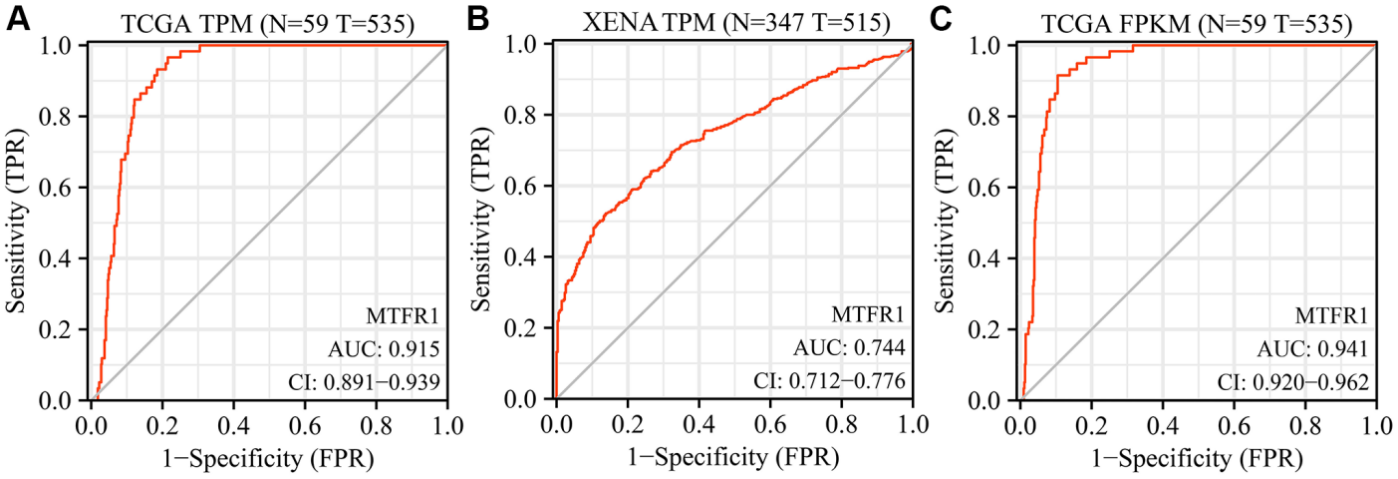
**Diagnostic values of MTFR1 in LAC from the TCGA and XENA databases.** (**A**, **B**) The data of TPM type; (**C**) The data of FPKM type. Abbreviations: LAC: lung adenocarcinoma; TCGA: The Cancer Genome Atlas; TPM: transcripts per million; FPKM: Fragments Per Kilobase Million.

**Figure 3:**
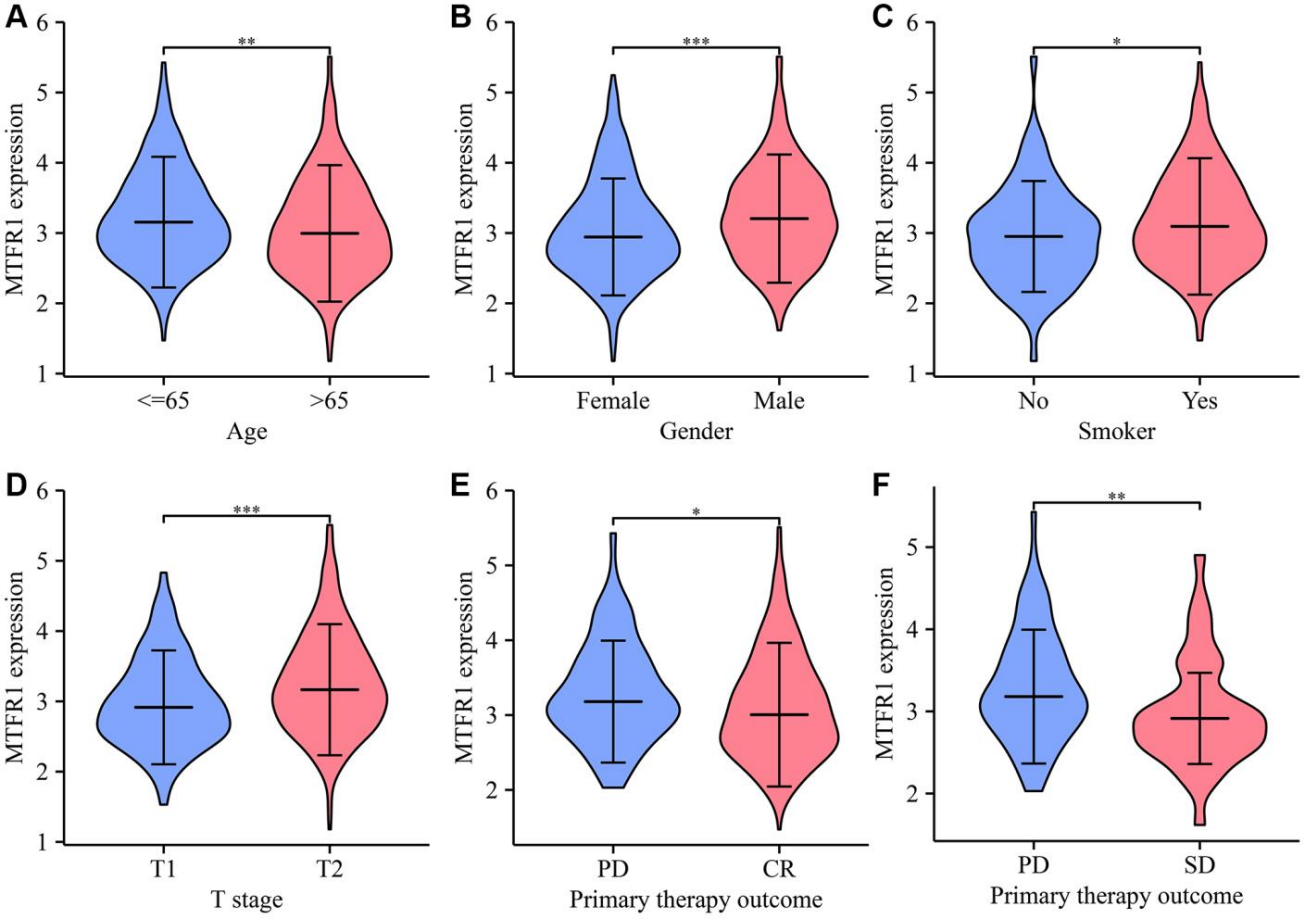
**The levels of MTFR1 have a significant difference in the clinicopathological features of patients with LAC.** (**A**) Age; (**B**) Gender; (**C**) Smoking; (**D**) T stage; (**E**, **F**) Treatment effects. Abbreviation: LAC: lung adenocarcinoma; ^*^P < 0.05; ^**^P < 0.01; ^***^P < 0.001.

Receiver operating characteristic (ROC) analysis revealed that the area under the curves of MTRF1 in normal tissues and LAC tissues were 0.915, 0.744, and 0.941 based on the TPM and FPKM data from TCGA and XENA database, respectively (Figure 2). This indicated that MTRF1 has significant diagnostic values in LAC. MTFR1 expression correlated with sex, age, primary pathological outcome, smoking and the T stage of patients with LAC (Figure 3). Furthermore, in the high- and low-expression groups of MTFR1, the expression levels of MTFR1 were significantly correlated with *T* stage, *N* stage, sex and age (Table 1).

**Table 1:** The relationship between MTFR1 expression and clinical features in LAC.

**Characteristic**	**Low expression of MTFR1**	**High expression of MTFR1**	***P***
T stage			0.001
T1	108 (20.3%)	67 (12.6%)	
T2	127 (23.9%)	162 (30.5%)	
T3	20 (3.8%)	29 (5.5%)	
T4	11 (2.1%)	8 (1.5%)	
N stage			0.035
N0	175 (33.7%)	173 (33.3%)	
N1	54 (10.4%)	41 (7.9%)	
N2	28 (5.4%)	46 (8.9%)	
N3	2 (0.4%)	0 (0%)	
M stage			0.631
M0	170 (44%)	191 (49.5%)	
M1	10 (2.6%)	15 (3.9%)	
Pathologic stage			0.470
Stage I	154 (29.2%)	140 (26.6%)	
Stage II	60 (11.4%)	63 (12%)	
Stage III	37 (7%)	47 (8.9%)	
Stage IV	11 (2.1%)	15 (2.8%)	
Gender			< 0.001
Female	163 (30.5%)	123 (23%)	
Male	104 (19.4%)	145 (27.1%)	
Age			0.035
≤65	115 (22.3%)	140 (27.1%)	
>65	143 (27.7%)	118 (22.9%)	
Residual tumor			0.606
R0	166 (44.6%)	189 (50.8%)	
R1	6 (1.6%)	7 (1.9%)	
R2	3 (0.8%)	1 (0.3%)	
Smoker			0.293
No	42 (8.1%)	33 (6.3%)	
Yes	217 (41.7%)	229 (44%)	
OS event			0.026
Alive	184 (34.4%)	159 (29.7%)	
Dead	83 (15.5%)	109 (20.4%)	
DSS event			0.071
Alive	199 (39.9%)	180 (36.1%)	
Dead	51 (10.2%)	69 (13.8%)	
PFI event			0.202
Alive	162 (30.3%)	147 (27.5%)	
Dead	105 (19.6%)	121 (22.6%)	

Abbreviations: LAC: lung adenocarcinoma; OS: overall survival; DSS: disease-specific survival; PFI: progression-free interval.

### MTFR1 overexpression correlated with unfavourable prognosis in patients with LAC

**Figure 4:**
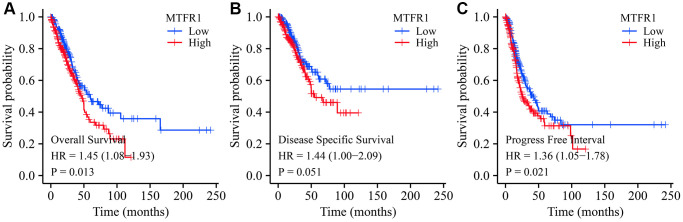
**Prognostic values of MTFR1 in LAC from the TCGA database.** (**A**) Overall survival; (**B**) DSS; (**C**) PFI. Abbreviations: LAC: lung adenocarcinoma; DSS: disease-free survival; PFI: progression-free interval; TCGA: The Cancer Genome Atlas.

**Figure 5:**
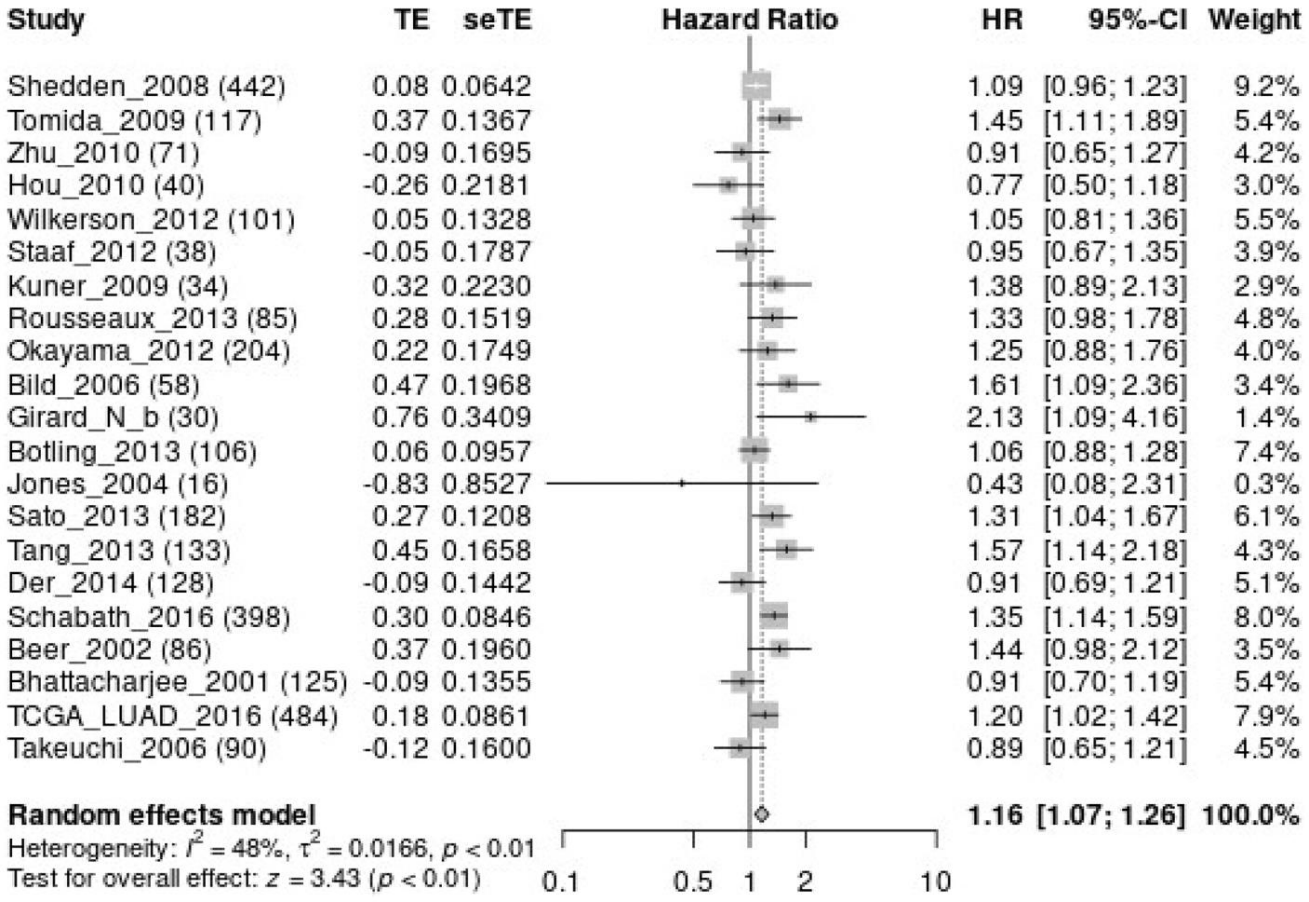
**MTFR1 over-expression was related to the overall survival time in patients with LAC using meta-analysis.** Abbreviations: LAC: lung adenocarcinoma.

**Figure 6:**
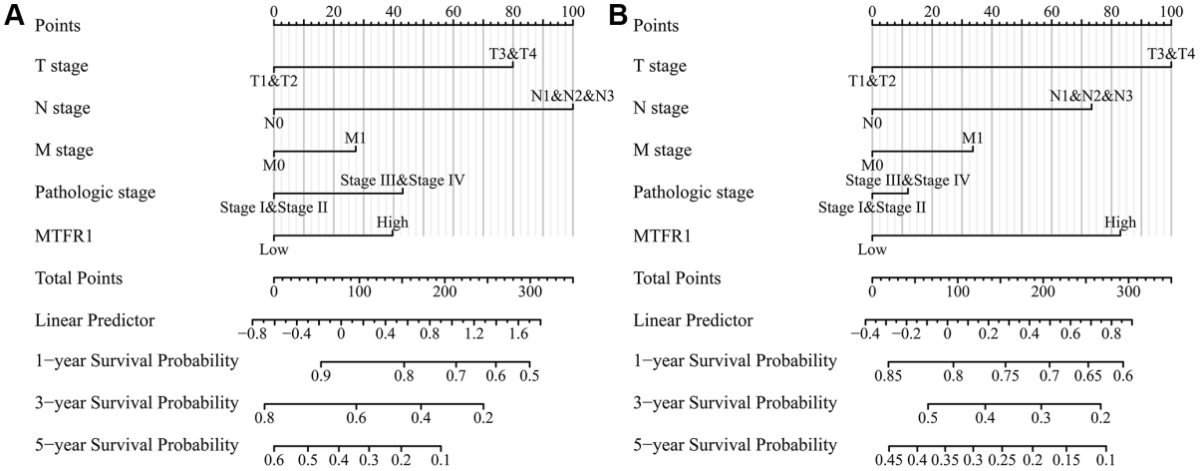
**MTFR1-related prognosis nomograms.** (**A**) Overall survival; (**B**) Progression-free interval.

Kaplan Meier (K-M) survival analysis revealed that increased MTFR1 expression was significantly associated with unfavourable OS, disease-specific survival (DSS), and progression-free interval (PFI) in patients with LAC based on the data of the FPKM type (Figure 4). Consistently, the Lung cancer explorer (LCE) database analysis also revealed that patients with high MTFR1 expression had an unfavourable prognosis (Figure 5). Additionally, univariate COX regression analysis revealed that T stage, N stage, M stage, pathological stage and MTFR1 overexpression were the risk factors for unfavourable OS (Table 2). Moreover, the T stage, N stage, M stage and pathological stage were the risk factors for unfavourable DSS (Table 3). Additionally, T stage, N stage, pathological stage and MTFR1 overexpression were the risk factors for unfavourable PFI (Table 4). The MTFR1-related nomograms in the OS and PFI were established based on the results of Cox analysis to assess the survival time of patients with cancer (Figure 6).

**Table 2:** Risk factors affecting OS in LAC.

**Characteristics**	***N***	**HR (95% CI)**	***P***
T stage	523		
T1	175	Reference	
T2	282	1.521 (1.068–2.166)	0.020
T3	47	2.937 (1.746–4.941)	<0.001
T4	19	3.326 (1.751–6.316)	<0.001
N stage	510		
N0	343	Reference	
N1	94	2.381 (1.695–3.346)	<0.001
N2	71	3.108 (2.136–4.521)	<0.001
N3	2	0.000 (0.000–Inf)	0.994
M stage	377		
M0	352	Reference	
M1	25	2.136 (1.248–3.653)	0.006
Pathologic stage	518		
Stage I	290	Reference	
Stage II	121	2.418 (1.691–3.457)	<0.001
Stage III	81	3.544 (2.437–5.154)	<0.001
Stage IV	26	3.790 (2.193–6.548)	<0.001
Gender	526		
Female	280	Reference	
Male	246	1.070 (0.803–1.426)	0.642
Age	516		
≤65	255	Reference	
>65	261	1.223 (0.916–1.635)	0.172
Smoker	512		
No	72	Reference	
Yes	440	0.894 (0.592–1.348)	0.591
MTFR1	526		
Low	262	Reference	
High	264	1.446 (1.081–1.935)	0.013

Abbreviations: LAC: lung adenocarcinoma; OS: overall survival.

**Table 3:** Risk factors affecting DSS in LAC.

**Characteristics**	***N***	**HR (95% CI)**	***P***
T stage	488		
T1	168	Reference	
T2	262	1.701 (1.085–2.668)	0.021
T3	43	2.846 (1.453–5.572)	0.002
T4	15	2.770 (1.061–7.230)	0.037
N stage	475		
N0	327	Reference	
N1	83	2.751 (1.808–4.185)	<0.001
N2	63	2.762 (1.698–4.493)	<0.001
N3	2	0.000 (0.000–Inf)	0.995
M stage	344		
M0	323	Reference	
M1	21	2.455 (1.269–4.749)	0.008
Pathologic stage	483		
Stage I	277	Reference	
Stage II	112	3.017 (1.931–4.715)	<0.001
Stage III	72	3.326 (2.028–5.457)	<0.001
Stage IV	22	4.632 (2.371–9.050)	<0.001
Gender	491		
Female	262	Reference	
Male	229	0.989 (0.687–1.424)	0.954
Age	481		
≤65	243	Reference	
>65	238	1.013 (0.701–1.464)	0.944
Smoker	477		
No	69	Reference	
Yes	408	1.040 (0.602–1.796)	0.889
MTFR1	491		
Low	245	Reference	
High	246	1.443 (0.998–2.086)	0.051

Abbreviations: LAC: lung adenocarcinoma; DSS: disease-specific survival.

**Table 4:** Risk factors affecting PFI in LAC.

**Characteristics**	***N***	**HR (95% CI)**	***P***
T stage	523		
T1	175	Reference	
T2	282	1.758 (1.276–2.422)	<0.001
T3	47	3.495 (2.199–5.556)	<0.001
T4	19	1.113 (0.444–2.791)	0.819
N stage	510		
N0	343	Reference	
N1	94	1.540 (1.118–2.122)	0.008
N2	71	1.498 (1.018–2.205)	0.040
N3	2	0.906 (0.127–6.485)	0.922
M stage	377		
M0	352	Reference	
M1	25	1.513 (0.855–2.676)	0.155
Pathologic stage	518		
Stage I	290	Reference	
Stage II	121	2.013 (1.478–2.742)	<0.001
Stage III	81	1.831 (1.257–2.669)	0.002
Stage IV	26	2.086 (1.189–3.657)	0.010
Gender	526		
Female	280	Reference	
Male	246	1.172 (0.901–1.526)	0.236
Age	516		
≤65	255	Reference	
>65	261	1.023 (0.784–1.335)	0.867
Smoker	512		
No	72	Reference	
Yes	440	0.968 (0.658–1.426)	0.870
MTFR1	526		
Low	262	Reference	
High	264	1.364 (1.048–1.775)	0.021

Abbreviations: LAC: lung adenocarcinoma; PFI: progress free interval.

### Inhibition of MTFR1 expression reduces LAC cell growth and migration

**Figure 7:**
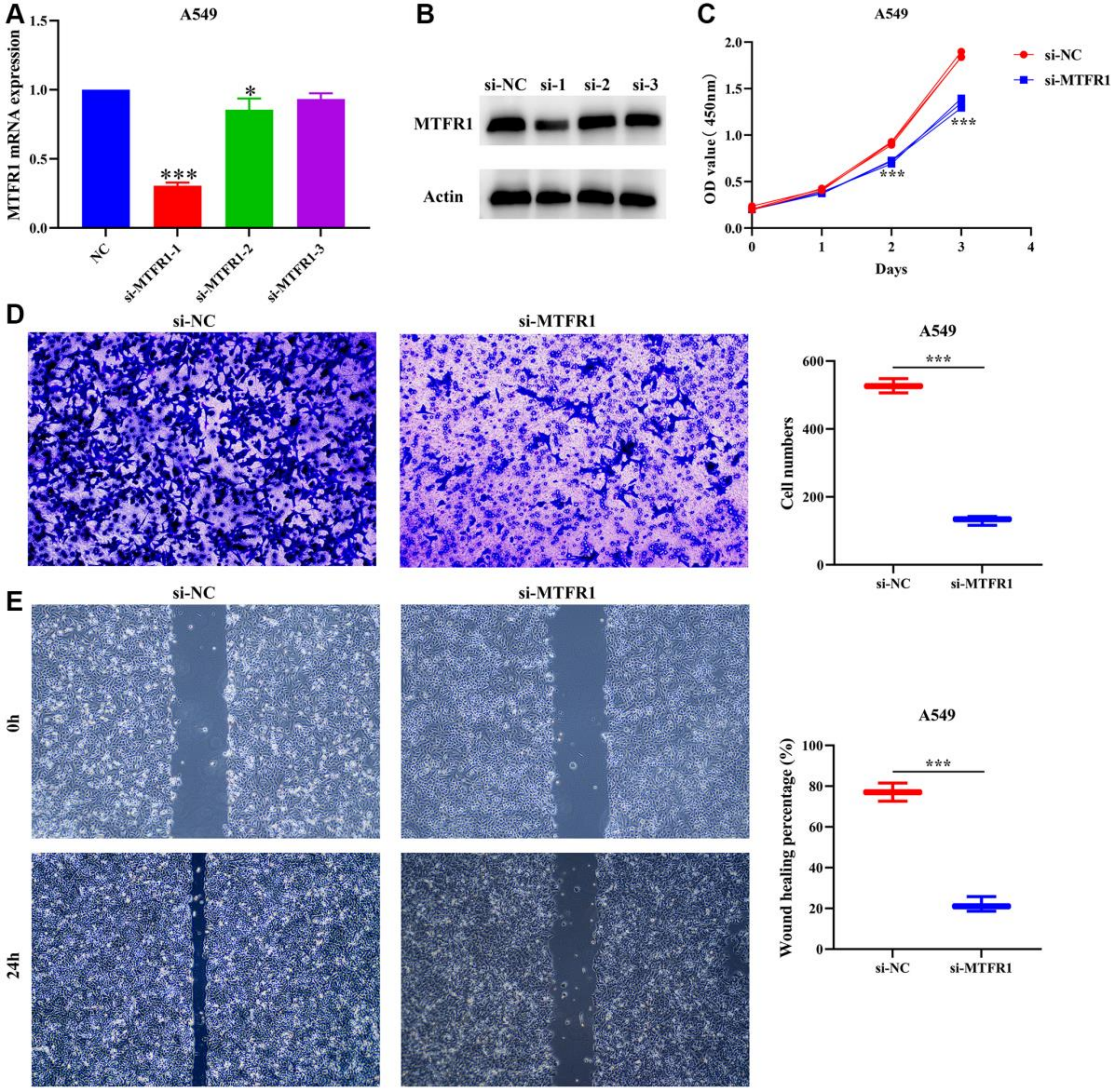
**Inhibition of MTFR1 expression could delay the proliferation, migration and invasion of A549 cells.** (**A**, **B**) Construction of cell model with downregulated MTFR1 expression; (**C**) Cell proliferation; (**D**) Cell invasion; (**E**) Cell migration. ^*^P < 0.05; ^***^P < 0.001.

A total of 391 MTFR1 co-expressed genes were positively related genes and 106 MTFR1 co-expressed genes were reversely related. Kyoto Encyclopedia of Genes and Genomes (KEGG) results showed that MTFR1 co-expressed genes were involved in the cell cycle, oocyte meiosis, DNA replication and other functions (Table 5). Figure 7A, 7B display the successfully constructed cell model. Additionally, the suppression of MTFR1 expression inhibited A549 cell proliferation, migration and invasion (Figure 7C–7E). Thus, these findings suggest that MTFR1 could reduce LAC cell growth and migration.

**Table 5:** The pathways of MTFR1 co-expressed genes.

**Term**	**Count**	***P***
hsa04110: Cell cycle	17	1.41E-07
hsa03013: Nucleocytoplasmic transport	12	1.07E-04
hsa04114: Oocyte meiosis	12	5.84E-04
hsa04914: Progesterone-mediated oocyte maturation	10	0.001291734
hsa05166: Human T-cell leukemia virus 1 infection	15	0.001904315
hsa03030: DNA replication	6	0.002212666
hsa04218: Cellular senescence	12	0.002431519
hsa05170: Human immunodeficiency virus 1 infection	13	0.009207311
hsa03430: Mismatch repair	4	0.020901599
hsa03008: Ribosome biogenesis in eukaryotes	8	0.02318999
hsa04940: Type I diabetes mellitus	5	0.024827891
hsa05161: Hepatitis B	10	0.025215676
hsa05145: Toxoplasmosis	8	0.026436598
hsa05014: Amyotrophic lateral sclerosis	17	0.027499011
hsa04658: Th1 and Th2 cell differentiation	7	0.032266665
hsa03420: Nucleotide excision repair	5	0.03313789
hsa05323: Rheumatoid arthritis	7	0.033774004
hsa04672: Intestinal immune network for IgA production	5	0.037832199
hsa04640: Hematopoietic cell lineage	7	0.043773992
hsa05012: Parkinson disease	13	0.044461525
hsa05152: Tuberculosis	10	0.045088011
hsa05310: Asthma	4	0.045634287

### Inhibition of MTFR1 expression promotes the sensitivity of LAC cells to cisplatin through p-AKT and p-ERK/p38 mechanisms

**Figure 8:**
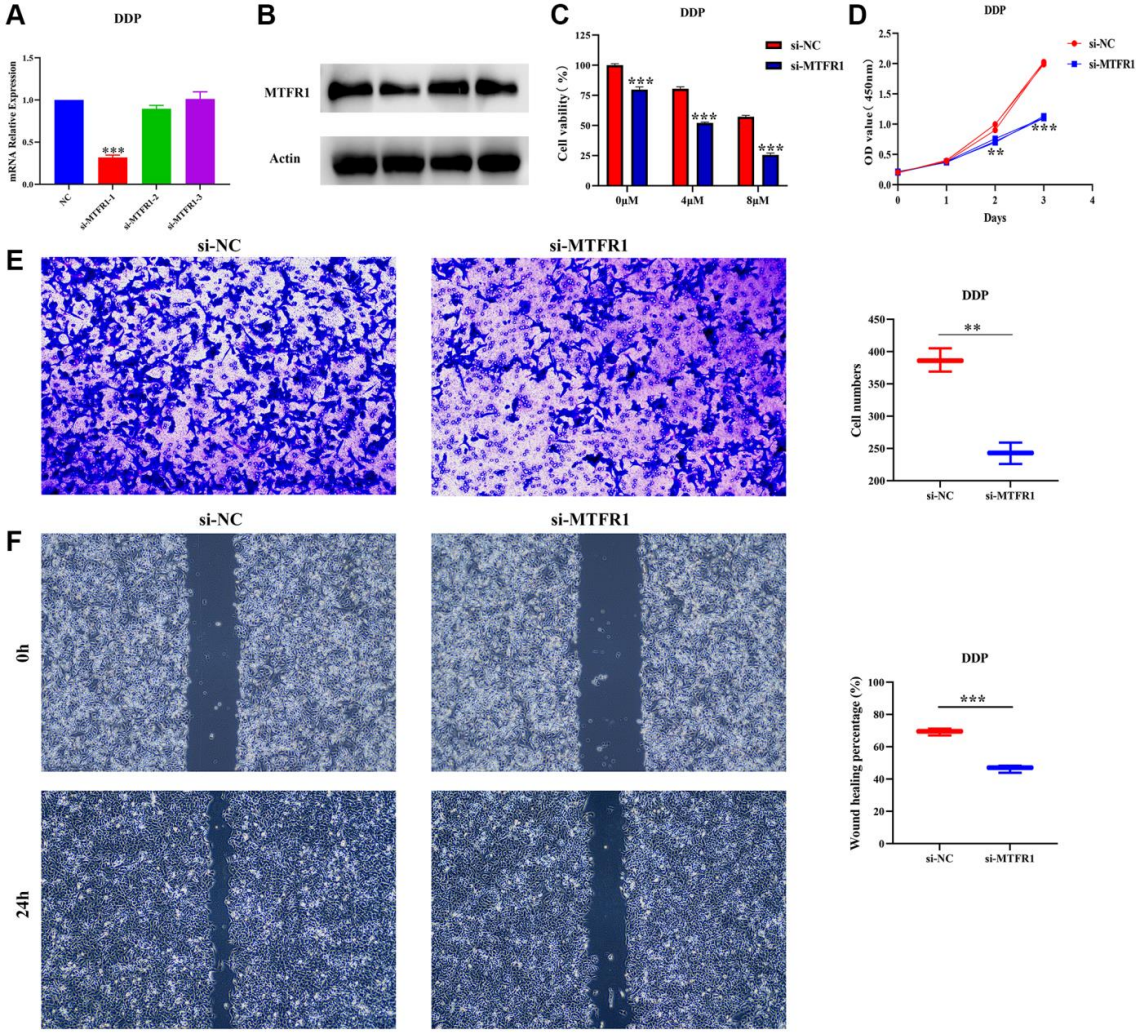
**Inhibition of MTFR1 expression could delay the progression and promote the sensitivity to cisplatin of A549/DDP cells.** (**A**, **B**) Construction of cell model with downregulated MTFR1 expression; (**C**) Cell viability; (**D**) Cell proliferation; (**E**) Cell invasion; (**F**) Cell migration. ^*^P < 0.05; ^**^P < 0.01; ^***^P < 0.001.

**Figure 9:**
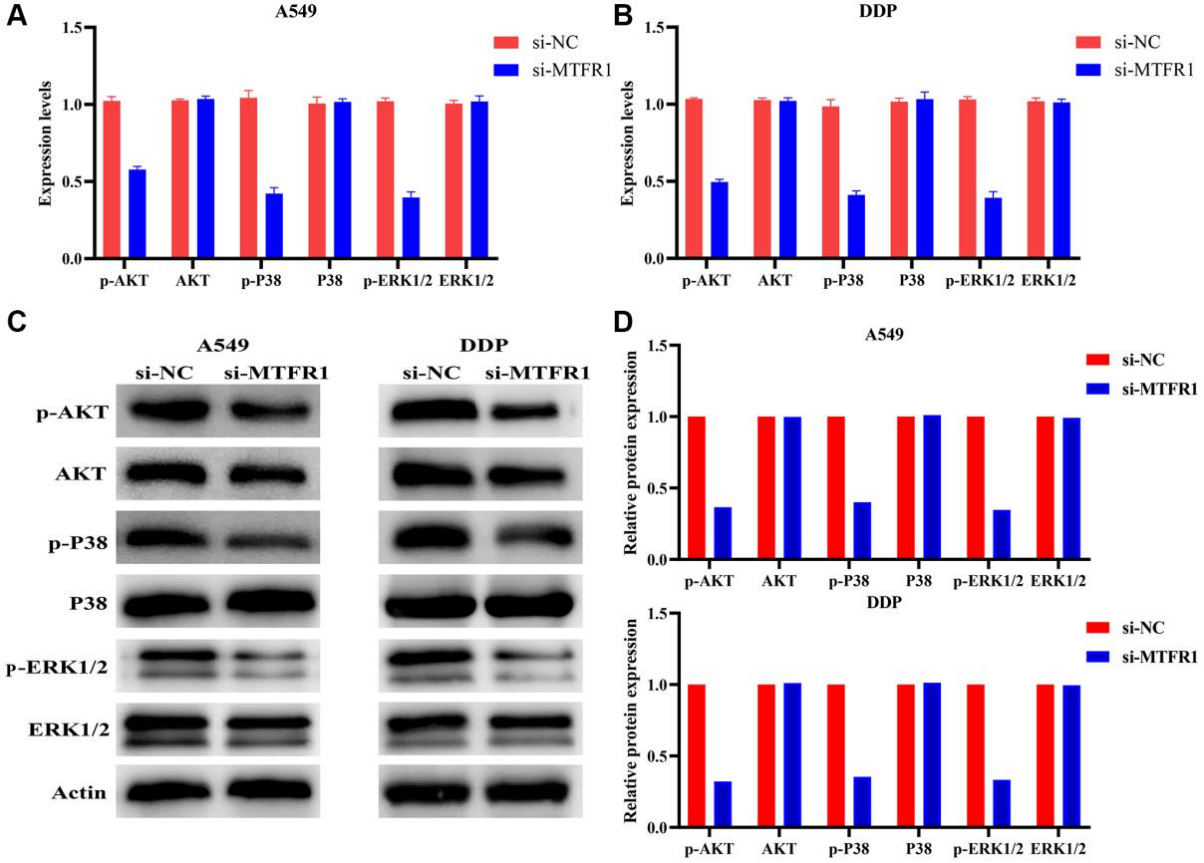
**Inhibition of MTFR1 expression could promote the sensitivity to cisplatin of LAC cells via the p-AKT and p-ERK/P38 signalling pathways.** (**A**) mRNA expression in A549 cells; (**B**) mRNA expression in A549/DDP cells; (**C**) Protein expression in A549 and A549/DDP cells; (**D**) Protein expression of statistics analysis. Abbreviation: LAC: lung adenocarcinoma.

In our cell model, cell Counting Kit-8 (CCK-8), migration and invasion assays revealed that the suppression of MTFR1 expression could inhibit A549/DDP cell proliferation, migration and invasion and promote the sensitivity of A549/DDP cells to cisplatin (Figure 8A–8F). Furthermore, the inhibition of MTFR1 expression downregulated the phosphorylation levels of extracellular regulated protein kinases (ERK), AKT serine/threonine kinase 1 (AKT) and P38 in A549 and A549/DDP cells, whereas the effects on the ERK, AKT and P38 were not significant, as shown using quantitative reverse transcription polymerase chain reaction (qRT-PCR) and western blotting (Figure 9). This suggests that MTFR1 expression promotes cell progression and the drug resistance of LAC cells to cisplatin through p-AKT and p-ERK/P38 mechanisms.

### MTFR1 expression was correlated with LAC immune infiltration

**Figure 10:**
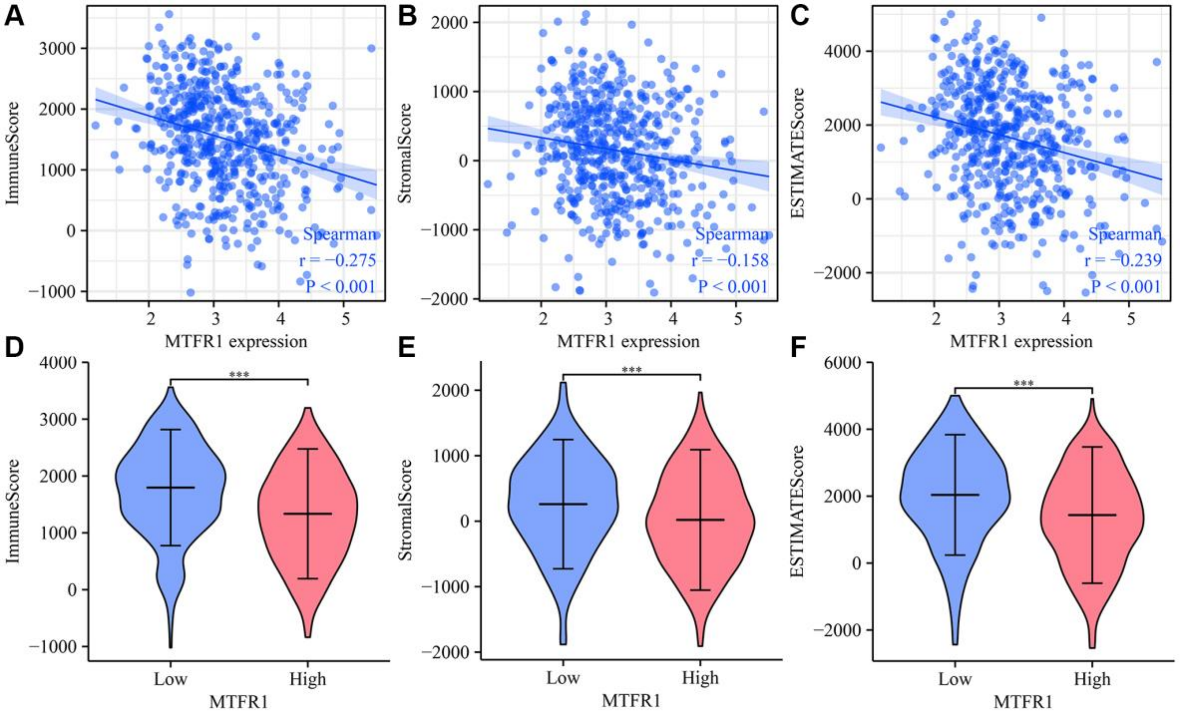
**MTFR1 expression correlated with the LAC immune microenvironment.** (**A**) Immune score; (**B**) Stromal score; (**C**) ESTIMATE score (**D**–**F**) Stromal, immune and ESTIMATE scores were statistically significant in high- and low-MTFR1 groups. Abbreviation: LAC: lung adenocarcinoma.

**Figure 11:**
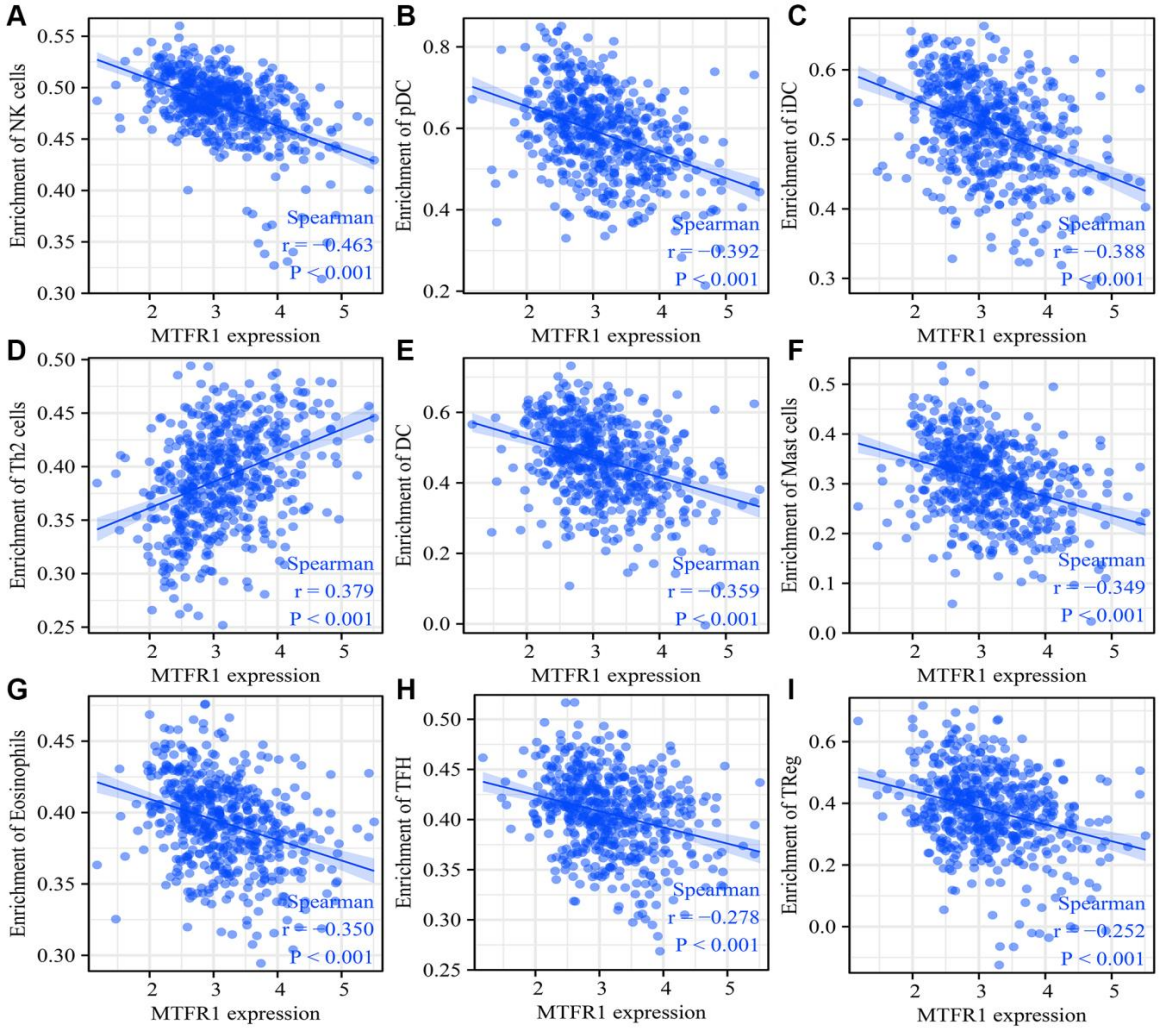
**MTFR1 expression correlated with the LAC immune cells.** (**A**) Natural killer cells; (**B**) pDCs; (**C**) IDC; (**D**) Th2 cells; (**E**) DCs; (**F**) Mast cells; (**G**) Eosinophils; (**H**)TFH; (**I**) Treg. Abbreviation: LAC: lung adenocarcinoma.

**Figure 12:**
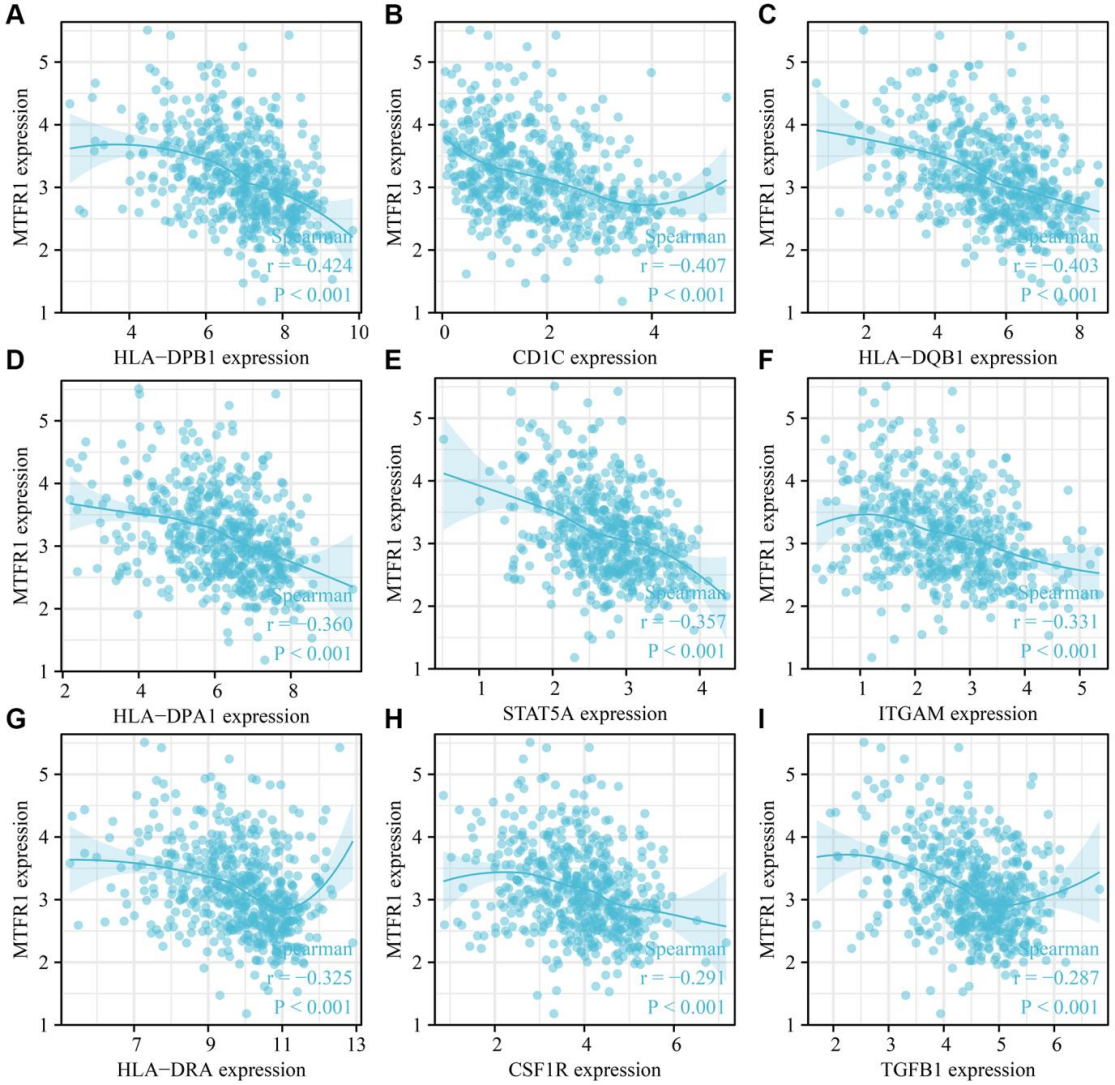
**MTFR1 expression correlated with the LAC immune cell markers.** (**A**) HLA-DPB1; (**B**) CD1C; (**C**) HLA-DQB1; (**D**) HLA-DPA1; (**E**) STAT5A; (**F**) ITGAM; (**G**) HLA-DRA; (**H**) CSF1R; (**I**) TGFB1. Abbreviation: LAC: lung adenocarcinoma.

MTFR1 overexpression was negatively correlated with stromal score (r = −0.158), immune score (r = −0.275) and ESTIMATE score (r = −0.239) (Figure 10A–10C). Stromal score, immune score and ESTIMATE score were statistically significant in the high- and low-MTFR1 expression groups (Figure 10D–10F). Moreover, MTFR1 expression levels were also significantly correlated with natural killer (NK) cells, pDCs, IDC, Th2 cells, DCs, eosinophils, mast cells, TFH, Treg, Th1 cells, macrophages, CD56 bright NK cells, Tgd, aDC, CD8 T cells, neutrophils, cytotoxic cells, T cells, Th17 cells and B cells (Figure 11 and Supplementary Figure 2). Additionally, B cells, aDC, Th2 cells and other immune cells were statistically significant in the high- and low-MTFR1 expression groups (Supplementary Figure 3). MTFR1 overexpression significantly correlated with LAC immune cell markers (such as HLA-DPB1, HLA-DQB1, HLA-DPA1, STAT5A, ITGAM, HLA-DRA, PD-1 and others) (Figure 12 and Table 6).

**Table 6:** MTFR1 expression levels correlate with immune molecules of LAC.

**Gene**	**Cor**	***P***	**Gene**	**Cor**	***P***
CD8A	0.003	0.950	HLA-DQB1	−0.403	<0.001
CD8B	0.024	0.578	HLA-DRA	−0.325	<0.001
CD3D	−0.123	0.004	HLA-DPA1	−0.360	<0.001
CD3E	−0.223	<0.001	CD1C	−0.407	<0.001
CD2	−0.167	<0.001	NRP1	0.068	0.118
CD19	−0.139	0.001	ITGAX	−0.279	<0.001
CD79A	−0.118	0.006	TBX21	−0.201	<0.001
CD86	−0.112	0.010	STAT4	−0.163	<0.001
CSF1R	−0.291	<0.001	STAT1	0.115	0.008
CCL2	−0.166	<0.001	IFNG	0.113	0.009
CD68	−0.269	<0.001	TNF	−0.153	<0.001
IL10	−0.013	0.770	GATA3	−0.187	<0.001
NOS2	−0.064	0.137	STAT6	−0.275	<0.001
IRF5	−0.231	<0.001	STAT5A	−0.357	<0.001
PTGS2	0.122	0.005	IL13	−0.099	0.022
CD163	−0.058	0.177	BCL6	−0.138	0.001
VSIG4	−0.115	0.008	IL21	0.068	0.115
MS4A4A	−0.079	0.068	STAT3	−0.039	0.362
CEACAM8	−0.235	<0.001	IL17A	0.030	0.489
ITGAM	−0.331	<0.001	FOXP3	−0.267	<0.001
CCR7	−0.269	<0.001	CCR8	−0.063	0.143
KIR2DL1	−0.059	0.173	STAT5B	−0.136	0.002
KIR2DL3	−0.029	0.497	TGFB1	−0.287	<0.001
KIR2DL4	0.138	0.001	PDCD1	−0.151	<0.001
KIR3DL1	−0.001	0.989	CTLA4	−0.097	0.026
KIR3DL2	−0.023	0.603	LAG3	−0.155	<0.001
KIR3DL3	0.096	0.026	HAVCR2	−0.123	0.005
KIR2DS4	−0.080	0.064	GZMB	0.103	0.017
HLA-DPB1	−0.424	<0.001			

Abbreviations: LAC: lung adenocarcinoma.

## DISCUSSION

Abnormal mitochondrial mitosis has been associated with tumour development [9, 17, 18]. For instance, Wang et al. reported that β-asarone, a natural compound with low toxicity, has anti-cancer activities that include mitochondria-linked apoptosis. β-asarone could inhibit the Wnt/β-catenin signalling transduction, reduce cell viability and facilitate mitochondria-related apoptosis in lung cancer cells. Moreover, β-asarone inhibited the viability of lung cancer cells in a dose-dependent manner and significantly inhibited the migration, invasion and adhesion of lung cancer cells [17]. Several studies have described various roles for MTFR1 in the mitochondrial division [11, 13, 16, 19, 20]. For instance, Wang et al. reported that, in oral squamous cell carcinoma extracapsular spread, MTFR1 could be regarded as an indicator of the prognosis of patients without lymph node metastasis [11]. However, to date, there have been no reports on the roles of MTFR1 in drug sensitivity and immunity in LAC. This study revealed that MTFR1 was significantly overexpressed in LAC tissues, and elevated MTFR1 expression was correlated with the sex, age, and T stage in patients with LAC. ROC results revealed that MTRF1 has significant diagnostic values in LAC. K-M survival analysis and meta-analysis revealed that MTFR1 overexpression was associated with OS, DSS, and progression-free interval (PFI) in patients with LAC. COX regression analysis revealed that MTFR1 overexpression was the risk factors for unfavourable OS and PFI. This indicated that MTFR1 overexpression was a prognostic risk factor for LAC patients.

Cell proliferation is associated with the mechanisms governing the cell cycle. Dysregulated cell cycling leads to the abnormal regulation of cell proliferation and can induce excessive increases in cell number, which leads to unfavourable prognosis in patients with tumours [12, 21, 22]. For example, mini-chromosome maintenance protein 10 (MCM10) strongly compensated for DNA replication pressure and promoted genome replication in S-phase cancer cells, which was more pronounced in cancer stem cell-like cells (CSC) [21]. FLAP endonuclease 1 (FEN1) was a structure-specific nuclease that plays a role in DNA replication and repair. Interference with FEN1 expression could kill defective human cells and induce DNA damage responses in sensitive and resistant cell lines [22]. These results indicate that cell cycle and DNA replication play an important role in cancer progression. Now, Wang et al. reported that MTFR1 could inhibit mitochondrial fission and induce apoptosis [11]. Similarly, Li et al. reported that MTFR1 was overexpressed in LAC and could promote LAC cell growth and migration [16]. Preliminary evidence suggested that MTFR1 was involved in cancer progression as a carcinogenic gene. In our study, MTFR1 co-expressed genes were involved in the cell cycle, DNA replication, cell differentiation and others, and interfering with MTFR1 expression could inhibit the proliferation, migration, and invasion of A549 and A549/DDP cells, and promote cell apoptosis and sensitivity to cisplatin through p-AKT and p-ERK/p38 mechanisms. These results were consistent with previously reported results, further validating that MTFR1 plays an important role in the drug resistance of LAC.

Cancer and the tumour microenvironment are inseparable [23–26]. Wang et al. observed a higher proportion of CD8 T cells in advanced ovarian cancer (OC) using immunohistochemistry. The degree of matrix CD8 T cell infiltration was the positive correlation between FoxP3 Treg cell infiltration and histological grade. Notably, the migration and invasion of OC cells and ascites-derived OC cells increased after co-culture with CD8 T cells [25]. Reches et al. reported that the binding of T cell immunoreceptor with Ig and ITIM domains (TIGIT) and nectin cell adhesion molecule 4 (Nectin4), a new ligand of TIGIT, inhibits the activity of NK cells. Furthermore, the blocked Nectin4 antibody can enhance tumour lethality *in vitro* and *in vivo* [26]. In keeping with the importance of tumour-associated immune cells, our data revealed that MTFR1 expression also correlated with LAC stromal cells and immune cells, as well as immune cell markers, indicating that MTFR1 might play a vital role in LAC progression and immune escape.

MTFR1 was identified to have the important biological roles in LAC progression using bioinformatics and cell experiments to provide a new candidate molecule for the treatment of patients with LAC. However, the present study has certain limitations. The proliferation, migration, invasion and drug sensitivity of each group of cancer cells after activating p-AKT or p-ERK expression in the cell models that interfered with MTFR1 expression were not examined and should be investigated, and the MTFR1 expression in A549 and A549/DDP cells would be detected in the future. In conclusion, MTFR1 overexpression correlated to the diagnostic value, sex, age, primary therapy outcome, smoking, T stage, prognosis and immune infiltration of patients with LAC. Moreover, MTFR1 participates in cell growth and migration and promotes the resistance of LAC cells to cisplatin via p-AKT and p-ERK/P38 signalling pathways. Thus, it can be considered a potential therapeutic target for the unfavourable prognosis of patients with LAC.

## MATERIALS AND METHODS

### TCGA and XENA databases

In May 2022, the genomic data of 535 cases of LAC and 59 cases of normal lung tissues of FPKM and TPM types were acquired from TCGA database and, the genomic data of 347 cases of normal lung tissues and 515 cases of LAC tissues of TPM types from XENA database. The gene expression data of 57 normal lung and LAC tissue samples, wherein the data belonged to the same 57 patients, were obtained and MTFR1 expression levels were investigated. The clinical data of patients with LAC from TCGA were screened. Furthermore, MTFR1 expression data and clinical data were combined to investigate the potential of MTFR1 in the clinicopathological characterisation and prognosis of patients with LAC.

### The expression levels and clinical values of MTFR1 in LAC

The diagnostic value of MTFR1 in normal lung tissues and cancer tissues derived from the TCGA and XENA databases was identified using ROC analysis [27]. The expression of MTFR1 in each group was investigated according to the clinical pathological characteristics of patients. Additionally, the relationship between MTFR1 expression level and survival time, disease-specific survival and PFI of patients with LAC were determined using survival analysis after grouping by MTFR1 expression median value. Moreover, in the LCE database, a meta-analysis was conducted using data from the TCGA and GEO databases to investigate the expression and prognostic value of MTFR1 in LAC [28]. The clinical data of patients from TCGA were collated, and the factors affecting the prognosis of patients with LAC were analysed using univariate COX regression analysis. Subsequently, the MTFR1-related nomograms were constructed.

### MTFR1 co-expressed genes

Spearman correlation coefficient (r) has been used to predict the relationship between two genes [29]. MTFR1 co-expressed genes in LAC tissues were filtrated based on *P* < 0.001 and the absolute values of coefficient >0.4, resulting in identifying the strongly related genes.

### Signalling mechanisms of MTFR1 co-expressed genes

KEGG analysed the biological functions and mechanisms of the genes involved [30]. The signalling pathways of MTFR1 co-expressed genes were authenticated using KEGG [31], with the cut-off criterion as *P* < 0.05, which denoted statistical significance.

### Cell culture and construction of cell models

Lung cancer cells A549 and A549/DDP were cultured in 10% foetal bovine serum (Gibao, USA) and 1% penicillin and streptomycin RPMI-1640 medium (Gibao, USA) and incubated in 5% CO_2_ at 37°C. Additionally, appropriate concentrations of cisplatin were added to A549/DDP cells. The A549 and A549/DDP cells were plated in 6-well plates at a 5 × 10^6^ concentration. When cell density reached 70%, the media was changed to the serum-free medium with the siRNAs [32]. The siRNA interference sequences of MTFR1 were the GCUGGAUUAAGCGCCUAAUTT (siRNA-1), CACUGCAGGUGACUUAGAUTT (siRNA-2), and CUGCUUGAUGAUUAATT (siRNA-3). The expression of MTFR1 was verified using qRT-PCR and western blotting at 24 h.

### qRT-PCR

Total RNAs were extracted from the 6-well plates after transfection using 1 ml Trizol (Takara, Japan) reagent. MTFR1 cDNA was synthesised using the SureScript™ First-Strand cDNA synthesis kit (Genecopoeia, China) after RNA quantification and amplified using blazetaqtmsybr^®^ green qPCR mix 2.0 (Genecopoeia, China). Finally, the relative expression of MTFR1 was calculated using the 2^−ΔΔCT^ method. Primers for β-actin, p-AKT, AKT, p-P38, P38, p-ERK1/2, ERK1/2 and MTFR1 were obtained from Genecopoeia. The experiment was repeated three times.

### Western blotting

Total proteins were extracted using RIPA lysate and quantified using BCA protein quantification. Proteins were separated using 10% SDS-PAGE and transferred onto polyvinylidene fluoride (PALL, USA) membranes [33, 34]. After blocking with 5% non-fat dry milk, the membranes were incubated with 1:1000 MTFR1, p-AKT, AKT, p-P38, P38, p-ERK1/2 and ERK1/2 antibodies overnight at 4°C and followed by 1:1000 secondary antibodies for 1 h at room temperature. After TBST washing, the membranes were visualised. The experiment was repeated three times.

### Cell counting kit-8 (CCK-8)

When the A549 and A549/DDP cells were at the logarithmic phase, 2500 cells/well were seeded in 96-well plates, placed in 5% CO_2_ and incubated at 37°C. Following this, 10 ul/well CCK-8 reagent was added into the 96-well plates at 0 h, 24 h, 48 h, 72 h and 96 h and incubated at 5% CO_2_, 37°C for 1 h to detect cell viability and drug sensitivity. The absorbance values of the cancer cells were detected at 450 nm using a microplate reader. The experiment was repeated thrice.

### Cell migration and invasion

A549 and A549/DDP cells at approximately 90% confluence after transfection were scratched with 0.2 ml sterile pipette heads, and the medium in the 6-well plates was changed to serum-free medium after phosphate-buffered saline (PBS) washing. Subsequently, the cancer cells were photographed. The migration distances of the cells in the two groups were observed in real time and photographed under a microscope, and the degree of migration was subsequently calculated. The model cell concentration was adjusted as follows: 200 μl cell suspension was added into the upper chamber of the Transwell membrane and 600 μl complete medium was added into the lower chamber. The no migrated cancer cells on the Transwell membrane were washed thrice using PBS after 5% CO_2_ and 37°C incubation for 24 h. The cells were subsequently stained using a cell chamber Staining Kit (Biosharp, China) and photographed under a microscope. The experiment was repeated thrice.

### Immune microenvironment analysis

ESTIMATE and single sample gene set enrichment analysis (ssGSEA) analysis were the bioinformatics algorithm that calculates the composition of immune cells, immune, ESTIMATE and stromal scores [35, 36]. The ratio of immune cells in LAC tissues was explored using ssGSEA analysis [35]. The immune, ESTIMATE and stromal scores were statistically analysed using the ESTIMATE algorithm [35, 36]. The relationship between MTFR1 expression and LAC immune infiltration was investigated using Spearman correlation analysis.

### The relationship between MTFR1 expression and immune cell markers

The data of immune cell markers and MTFR1 expression were extracted from the data of the LAC tissues in TCGA using Perl. The data were then sorted out, and correlation analysis was used to investigate the relationship between MTFR1 expression level and CD8A, CD8B, PDCD1 and other immune cell markers.

### Statistical analysis

The Wilcoxon signed-rank test and chi-square test were applied to investigate the relationship between MTFR1 expression and clinicopathological characteristics in LAC. K-M survival analysis was applied to authenticate the correlation between MTFR1 expression and poor prognosis in patients with LAC. The *t*-test (mean ± standard deviation) was used to identify whether the cell proliferation and migration experiments were statistically significant. *P* < 0.05 was considered statistically significant.

### Data availability statement

The data is available from the databases and the experimental data could be obtained from the corresponding author.

## Supplementary Materials

Supplementary Figures
