# Supplementary material for: Overexpression of MTFR1 promotes cancer progression and drug-resistance on cisplatin and is related to the immune microenvironment in lung adenocarcinoma
Source: Aging (Albany NY). 2024 Jan 2;16(1):66–88. doi: 10.18632/aging.205338 (PMC10817379; doi:10.18632/aging.205338)
Supplement: Supplementary Figures [file aging-16-205338-s001.pdf]

SUPPLEMENTARY FIGURES

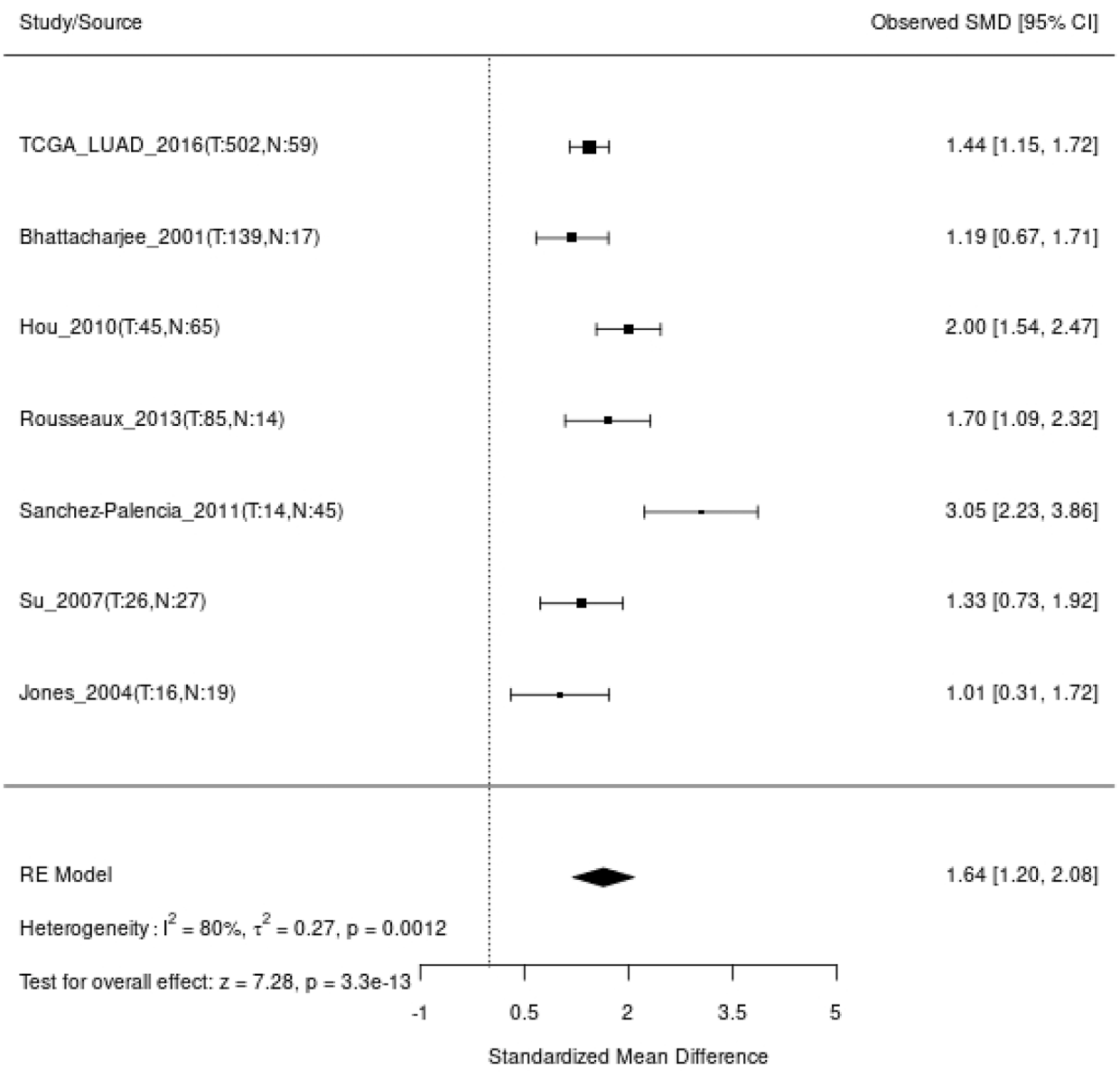

Supplementary Figure 1. MTFR1 expression in lung tissues using meta-analysis.

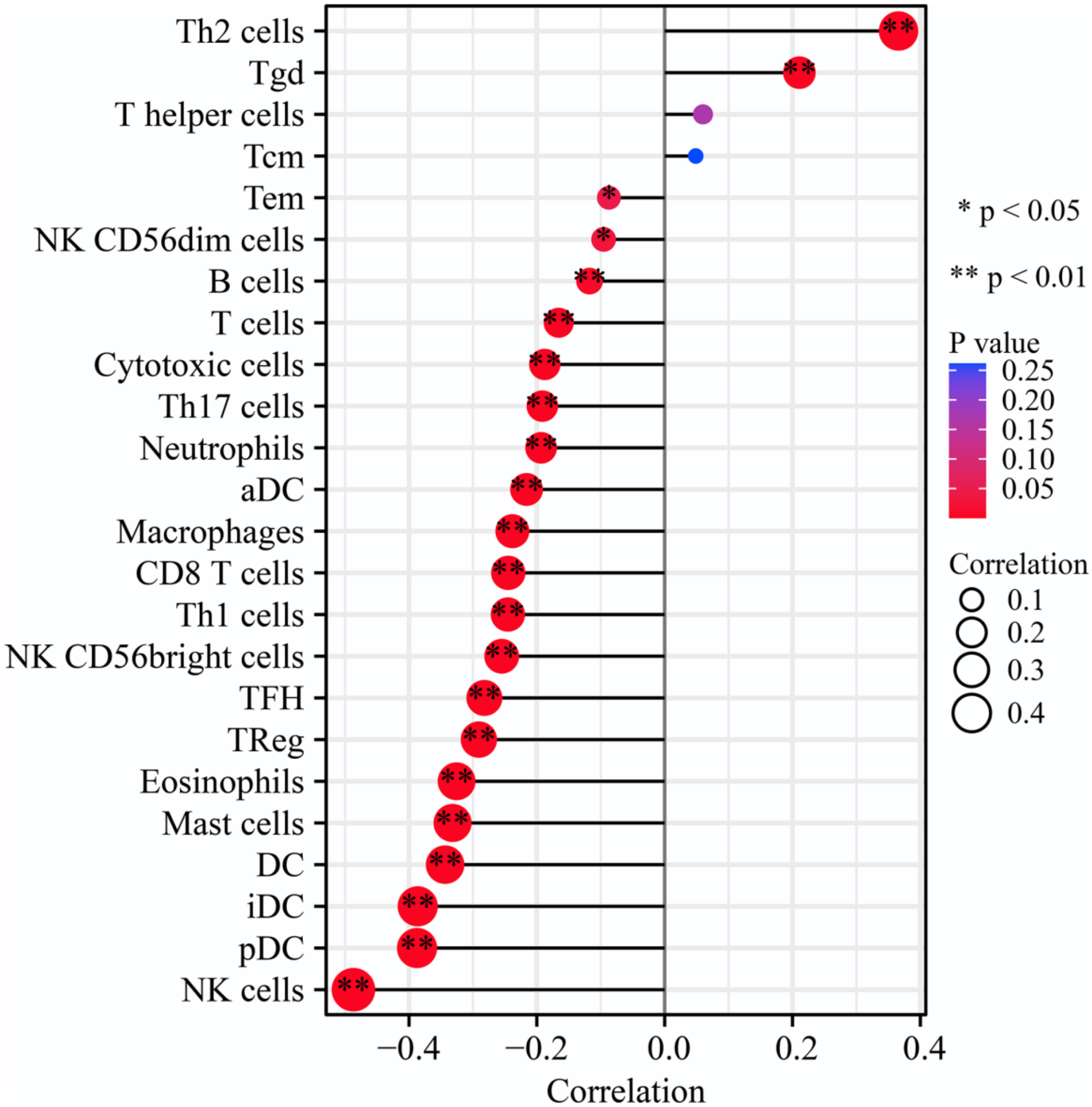

Supplementary Figure 2. MTFR1 expression was related to LAC immune cells in TCGA database.

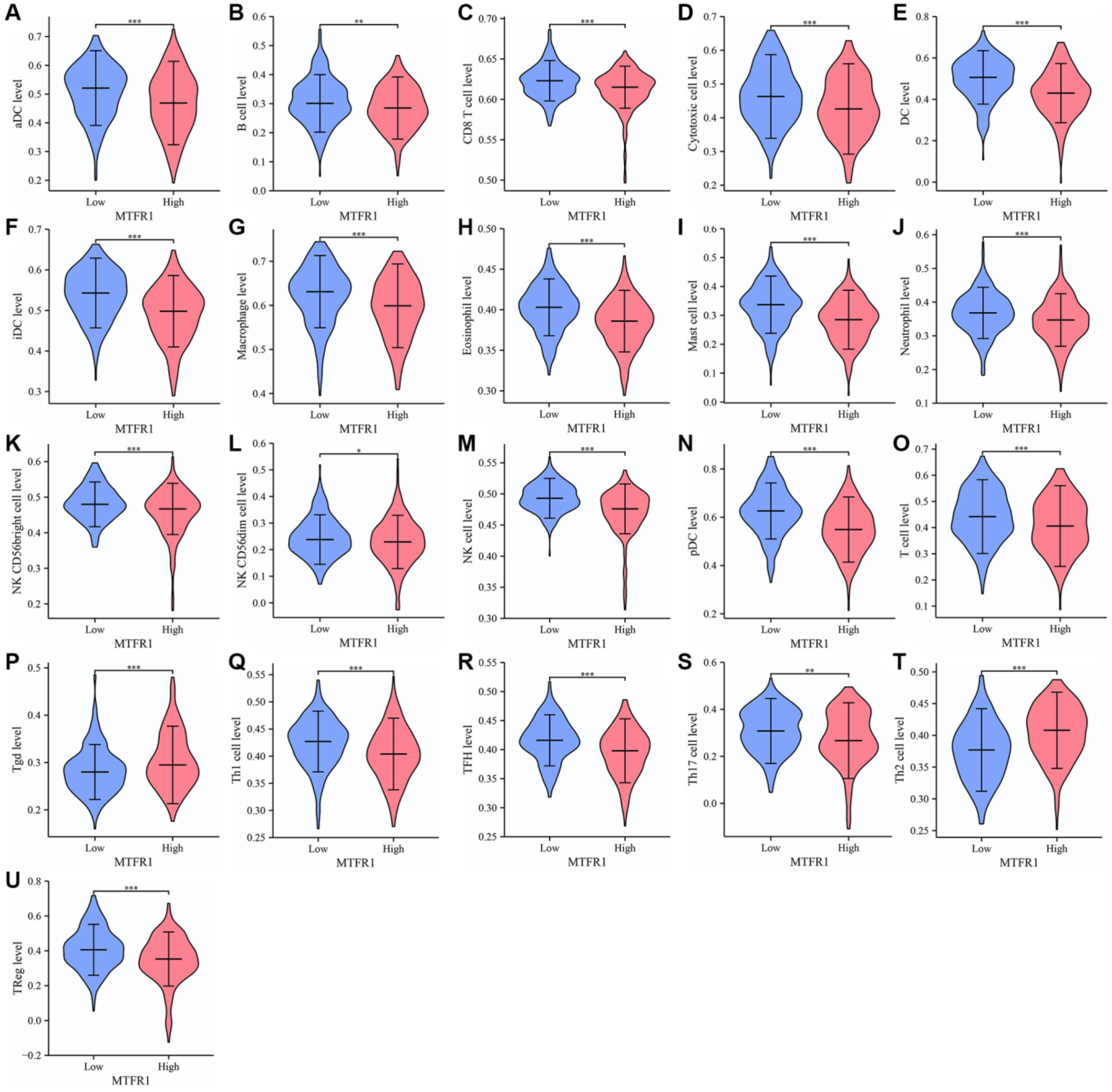

Supplementary Figure 3. The immune cells levels in high- and low- MTFR1 expression.
